# *In vitro* and *in vivo* activity of melflufen (J1)in lymphoma

**DOI:** 10.1186/s12885-016-2299-9

**Published:** 2016-04-04

**Authors:** Maryam Delforoush, Sara Strese, Malin Wickström, Rolf Larsson, Gunilla Enblad, Joachim Gullbo

**Affiliations:** Department of Immunology, Genetics and Pathology, Rudbeck Laboratory, Uppsala, Sweden; Department of Medical Sciences, Section of Clinical Pharmacology, Uppsala University Hospital, Uppsala, Sweden; Department of Women’s and Children’s Health, Karolinska Institutet, Childhood Cancer Research Unit, Stockholm, Sweden

**Keywords:** J1, Melflufen, Prodrug, Cancer therapeutics, Alkylating agents

## Abstract

**Background:**

Melphalan has been used in the treatment of various hematologic malignancies for almost 60 years. Today it is part of standard therapy for multiple myeloma and also as part of myeloablative regimens in association with autologous allogenic stem cell transplantation. Melflufen (melphalan flufenamide ethyl ester, previously called J1) is an optimized derivative of melphalan providing targeted delivery of active metabolites to cells expressing aminopeptidases. The activity of melflufen has compared favorably with that of melphalan in a series of in vitro and in vivo experiments performed preferentially on different solid tumor models and multiple myeloma. Melflufen is currently being evaluated in a clinical phase I/II trial in relapsed or relapsed and refractory multiple myeloma.

**Methods:**

Cytotoxicity of melflufen was assayed in lymphoma cell lines and in primary tumor cells with the Fluorometric Microculture Cytotoxicity Assay and cell cycle analyses was performed in two of the cell lines. Melflufen was also investigated in a xenograft model with subcutaneous lymphoma cells inoculated in mice.

**Results:**

Melflufen showed activity with cytotoxic IC_50_-values in the submicromolar range (0.011-0.92 μM) in the cell lines, corresponding to a mean of 49-fold superiority (*p* < 0.001) in potency vs. melphalan. In the primary cultures melflufen yielded slightly lower IC_50_-values (2.7 nM to 0.55 μM) and an increased ratio vs. melphalan (range 13–455, average 108, *p* < 0.001). Treated cell lines exhibited a clear accumulation in the G2/M-phase of the cell cycle. Melflufen also showed significant activity and no, or minimal side effects in the xenografted animals.

**Conclusion:**

This study confirms previous reports of a targeting related potency superiority of melflufen compared to that of melphalan. Melflufen was active in cell lines and primary cultures of lymphoma cells, as well as in a xenograft model in mice and appears to be a candidate for further evaluation in the treatment of this group of malignant diseases.

## Background

In their pioneering work on nitrogen mustards, Gilman and Philips [1] laid the foundation for modern cancer chemotherapy. These compounds were chemically unstable to hydrolysis and efforts were soon made to increase the stability without reduction of activity. Substitution with electron-withdrawing groups, e.g., a substituted phenyl ring, decreased the rate of aziridinium ion formation and reactivity of the nitrogen mustard [2]. In the early fifties the British scientists Bergel and Stock hypothesized that certain natural amino acids or peptides might, when modified by appropriate groups, show anti-tumor activity by interference with the nucleic acid or protein metabolism of malignant cells [3]. In this work the D, L, and DL-form of *p-bis*(2-chloroethyl)aminophenylalanine were synthesized and high activity of the L-form, but only slight activity of the D-form, was demonstrated in a Walker rat carcinoma model [4, 5]. The lead compound was later named “melphalan”, the name being derived from mustard-L-phenylalanine [6]. Intravenous melphalan has single-agent activity in a variety of human malignancies including for example breast cancer, ovarian cancer, testicular cancer and multiple myeloma [7–9]. It was also acknowledged early on, that in poor-prognosis patients with lymphoma, multiple myeloma, or neuroblastoma, high-dose melphalan-containing regimens (>140 mg/m^2^) yielded both high response rates and improved survival, despite considerable toxicity [10]. Melphalan is an important component in the high-dose conditioning chemotherapy regimen most used for lymphoms, BEAM (BCNU, etoposide, cytarabine and melphalan).

Malignant lymphomas are a group of tumors originating from cells of the lymphatic system. The lymphomas are classified according to the WHO-classification into B-cell lymphomas, T-cell lymphomas and Hodgkin lymphoma (HL) and further into many different entities. Of the B-cell lymphomas, diffuse large B-cell lymphoma (DLBCL) is the most common subtype. Systemic chemotherapy is the mainstay of therapy for all lymphomas and approximately 60–70 % of DLBCL patients are cured. In HL even higher cure rates are possible, and the chemotherapy may be combined with radiotherapy. Poor responders and/or early relapse patients at young age and good physical condition are often considered for high dose chemotherapy and stem cell transplantation. Still a significant proportion of lymphoma patients relapse, and there is a need for new drugs to further improve the results and reduce the toxicity of treatment [11, 12].

Melflufen (L-melphalanyl-p-L-fluorophenylalanine ethyl ester hydrochloride, previously called J1) is a derivative of the classical alkylating agent melphalan. Chemically melflufen may be described as the ethyl ester of a dipeptide consisting of melphalan and *para*-fluoro-L-phenylalanine. The drug is susceptible to hydrolysis by aminopeptidases, like aminopeptidase N (APN; also designated CD13) for which melflufen is a substrate [13], that are frequently expressed or overexpressed in tumor tissue [14], providing the molecule with a target directed delivery to cells [15]. It has been demonstrated that melflufen exposure to various malignant cells in vitro results in at least a 10–20 fold higher intracellular concentration of melphalan in comparison with direct treatment with equimolar doses of melphalan [13, 15, 16]. The result is a cytotoxic IC_50_-value of melflufen significantly lower than for melphalan (10-600-fold lower in various in vitro cell systems) [15, 17, 18]. The advantage of melflufen vs. melphalan has also been demonstrated in various hollow fiber and/or xenograft human tumor models in rodents [16, 19, 20].

Increased expression of various hydrolytic enzymes like peptidases, esterases and proteases has been described in several types of human malignancies, especially those characterized by fast-growing and aggressive phenotypes [21]. Among these enzymes the metalloproteinase APN has received substantial attention as a marker and mediator of the malignant phenotype, as well as a possible target for anticancerous chemotherapy [14]. It has recently been shown that APN is directly involved in targeted delivery of melflufen resulting in intracellular enrichment of melphalan and subsequent cell death [16, 22]. APN is commonly expressed in hematopoietic malignancies of myelomonocytic origin and has less commonly been described in lymphoid neoplasms. However, expression of APN has been described in anaplastic large cell lymphoma [23, 24]. In addition, the aminopeptidase inhibitor bestatin (Ubenimex) inhibits the proliferation of histiocytic lymphoma cell line U937 and induces morphological, cytochemical and functional differentiation into monocyte/macrophages [25]. Bestatin has also been evaluated in clinical trials showing therapeutic efficacy and survival benefit in diseases like acute myeloid leukemia (AML) and lymphomas [26].

This study was undertaken to characterize the activity of melflufen against lymphoma cells in vitro and in vivo.

## Methods

### Cell lines & cell culture

Cytotoxicity was assayed in a panel of twelve human lymphoma cell lines. A brief description of the cell lines is presented in Table 1. The identity of the cell lines was confirmed by department of Laboratory Medicine, Karolinska Institutet in collaboration with department of Oncology and Pathology, Cancer Center Karolinska, Karolinska Institutet, Stockholm, Sweden.

**Table 1:** List of cell lines used in the study

Cell line	ID	Cell type	Subtype	Resource	Reference	Authentication Y/N
DB	ACC539	DLBCL	GCB	DSMZ, Braunschweig, Germany	[34, 35]	Y
DOHH-2	ACC47	DLBCL	GCB	DSMZ, Braunschweig, Germany	[36]	N
HDLM-2	ACC 17	HL	GCB	DSMZ, Braunschweig, Germany	[37, 38]	N
KM-H2	ACC 8	HL	GCB	DSMZ, Braunschweig, Germany	[37, 39]	N
L-428	ACC 197	HL	GCB	DSMZ, Braunschweig, Germany	[37, 40]	N
OCI-LY3	ACC 761	DLBCL	ABC	DSMZ, Braunschweig, Germany	[41, 42]	Y
RC-K8	ACC561	DLBCL	ABC	DSMZ, Braunschweig, Germany	[41, 43]	Y
SU-DHL-6	ACC572	DLBCL	GCB	DSMZ, Braunschweig, Germany	[44, 45]	Y
SU-DHL-10	ACC576	DLBCL	GCB	DSMZ, Braunschweig, Germany	[44, 46]	Y
U-2932	ACC633	DLBCL	ABC	DSMZ, Braunschweig, Germany	[47, 48]	Y
U-2940	ACC634	DLBCL	PMBL	DSMZ, Braunschweig, Germany	[30]	Y
WSU-NHL	ACC58	DLBCL	GCB	DSMZ, Braunschweig, Germany	[31, 34]	N

All lymphoma and CCRF-CEM cells were cultured in RPMI 1640 cell growth medium (Sigma-Aldrich, St Louis, MO, USA) supplemented with 10–20 % heat-inactivated fetal calf serum, 2 mM glutamine, 100 U/mL penicillin, and 100 μg/mL streptomycin (all chemicals from Sigma Aldrich). Cells were grown at 37 °C in a humidified atmosphere containing 5 % carbon dioxide, split twice weekly and harvested in log-phase for experimental use.

Patient tumor cells from 16 patients with different lymphoma subtypes were also analyzed for in vitro sensitivity (Table 2). The samples were obtained by routine surgery, diagnostic biopsy or bone marrow/peripheral blood sampling. The use of patient samples was approved by the regional Ethics Committee of Uppsala University (Ns 2008/246 and 2014/233). Informed consent was waived. Lymphoma cells were isolated from bone marrow or peripheral blood by 1.007 g/ml Ficoll-Paque (Pharmacia Biotech, Uppsala) density gradient centrifugation [27]. Tumor tissue from solid samples was minced into small pieces and tumor cells were then isolated by collagenase dispersion followed by purification on Percoll (Kabi Pharmacia) or Ficoll density gradient centrifugation [28]. Cell viability was determined by trypan blue exclusion test and the proportion of tumor cells in the preparation was judged by inspection of May–Grunwald–Giemsa-stained cytospin preparations by a cytopathologist. Cell culture medium RPMI 1640 (supplemented as described above) was used throughout. In some cases, cells were cryopreserved in a medium containing 10 % dimethylsulfoxide (DMSO, Sigma-Aldrich) and 90 % inactivated calf serum by initial freezing for 24 h at −70 °C, followed by storage at −150 °C. Cryopreservation in this way does not affect drug sensitivity [29]. The characteristics of the primary cultures of human lymphoma cells are listed in Table 1.

**Table 2:** Characteristics of primary cells from lymphoma patients

Sample No.	Diagnosis	Status at analysis
1	Indolent lymphoma	Refractory
2	Indolent lymphoma	Refractory
3	MCL	Refractory
4	NHL nos	Refractory
5	T-LB	Primary
6	DLBCL	Refractory
7	NHL nos	Refractory
8	FL	Refractory
9	Lymphoma nos	Primary
10	NHL nos	Unknown
11	MCL	Refractory
12	T-PLL	Refractory
13	MCL	Refractory
14	DLBCLtr GC	Refractory
15	DLBCLtr GC	Refractory
16	B-cell lymphoma nos	Primary

*MCL* Mantle cell lymphoma, *NHL* Non-Hodgkin lymphoma, *T-LB* Lymphoblastlymphoma of T-cell type, *FL* Follicular lymphoma, *T-PLL* Prolymphocytic leukemia of T-cell type, *DLBCLtr GC* Diffuse large B-cell lymphoma transformed from follicular lymphoma of germinal centre subtype, *Nos* Not otherwise specified

### Drugs and chemicals

**Fig. 1:**
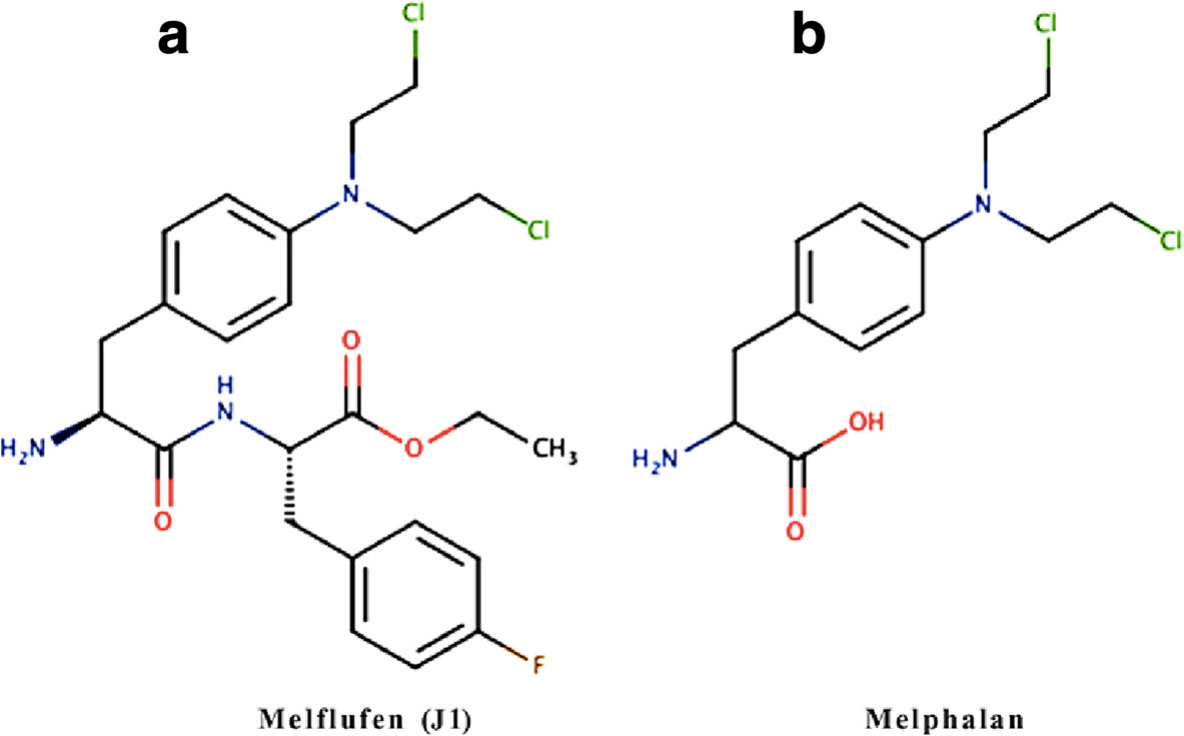
Chemical structure of melflufen (**a**) and melphalan (**b**)

Melphalan (Fig. 1a) was obtained as Alkeran® from the Swedish Pharmacy (Apoteket AB, Sweden), or bought as a pure chemical from Sigma Aldrich. Melflufen (Fig. 1b, kind gift from Oncopeptides AB, Stockholm, Sweden) was dissolved in DMSO and further diluted in sterile water or phosphate buffered saline (PBS; Sigma-Aldrich). All dilutions with water were made immediately prior to the start of the experiments to minimize the influence of mustard hydrolysis. The standard drugs vincristine, doxorubicin, etoposide, and cytarabine (all from Sigma), as well as 4-hydroxy-cyclophosphamide (4-HC, the active metabolite of cyclophosphamide, purchased from Niomech – IIT GmbH, Bielefeld, Germany) were dissolved in DMSO and diluted in PBS or sterile water prior to start of the experiments.

### The fluorometric microculture cytotoxicity assay FMCA

Cell viability after drug exposure (cytotoxic efficacy) was analyzed with the fluorometric microculture cytotoxicity assay (FMCA) which has been described in detail previously [30, 31]. The FMCA is a total cell kill assay performed in 96- or 384-well microtiterplates, and based on measurement of fluorescence generated from hydrolysis of fluorescein diacetate (FDA; Sigma-Aldrich). Cell lines were harvested in log-phase and seeded (45 μL/well) into 384-well microtiter plates (Nunc) at a concentration of 100,000 cells/ml using pipetting robot Biomek® 2000 (Beckman Coulter). The plates were incubated at 37 °C in humidified atmosphere containing 95 % air and 5 % CO_2_ for 24 h followed by the addition of drugs (2.5 nl portions/well of DMSO drug stock) using Echo 550 Liquid Handler (Labcyte). The plates were further incubated for 72 h. Patient lymphoma cells were handled similarly or seeded (45 μL/well, 40,000 cells/well) into drug containing (5 μL/well) 384-well microtiter plates. The plates were incubated at 37 °C, in humidified atmosphere containing 95 % air and 5 % CO_2_ for 72 h. Each drug concentration was tested in duplicates. Each 384-well microtiter plate had three columns without drugs serving as controls, and one column with medium served as blank. The cells were subsequently washed with PBS and supernatant aspirated leaving 10 μl in each well, before the addition of 50 μL, 10 μg/mL FDA. Plates were incubated for 60 min before measurement of fluorescence (485/538 nm for excitation and emission respectively) in a Fluostar Omega. The fluorescence generated is proportional to the number of cells in the well with an intact plasma membrane, and data are presented as Survival Index (SI %), defined as the mean fluorescence of the test wells expressed as a percentage of control cultures, with blank values subtracted.

Quality criteria for a successful assay included > 75 % starting viability (judged by Trypan blue exclusion), a control signal more than ten times the blank, and finally, a coefficient of variation in control and blank wells of < 30 %.

### Cell cycle distribution analysis

Cell cycle distribution analyses were performed on melflufen treated (0.1, 0.2 and 0.4 μM) and control cells from the KM-H2 and SU-DHL-10 cell lines in which the cytotoxic activity of melflufen during the 72 h FMCA assay was calculated as 0.92, 0.22, and 0.71 μM respectively (see Table 3). To minimize potential artifacts emanating from cells exiting the analyzed cell populations, e.g. due to early and/or extensive cell death, the cells were subjected to basal cell culture conditions for 40 h (preincubation) followed by 12, 24 and 48 h treatments with melflufen. Propidium iodide (PI) staining of DNA using Vindelöv’s technique was followed by quantitation with flow cytometry analysis. The percentage of cells in each cell cycle phase was determined with the ModFit LT software.

**Table 3:** Cytotoxic activity (as IC_50_ μM) of standard drugs and melflufen in the panel of cell lines

Drugs/Cell lines	Vincristine	Doxorubicin	4-HC	Etoposide	Cytarabine	Bortezomib	Melphalan	Melflufen
DB	0.013	0.052	25	2.4	30	0.0020	44	0.92
DOHH-2	0.030	0.050	22	2.3	9.6	0.0063	17	0.39
HDLM-2	0.0023	0.15	16	3.9	0.83	0.0031	9.3	0.088
KM-H2	0.0025	0.034	5.7	0.65	>100	0.0024	8.6	0.22
L-428	0.14	0.089	24	2.0	31	0.0044	9.4	0.73
Ly-3	0.0026	0.044	0.77	0.24	1.4	0.0028	0.52	0.011
RC-K8	0.14	0.059	21	2.7	8.7	0.0071	12	0.45
SU-DHL-6	0.0014	0.053	6.7	2.7	7.0	0.0023	38	0.42
SU-DHL-10	0.00026	0.19	17	49	0.080	0.0047	29	0.71
U-2932	0.0037	0.18	17	3.5	0.84	0.0038	19	0.52
U-2940	0.020	0.026	1.6	0.34	0.91	0.0052	9.8	0.12
WSU-NHL	0.0015	0.032	3.8	1.5	0.17	0.0028	6.9	0.077

### Effect of melflufen in DOHH-2 xenografted mice

The xenograft study was performed at Pipeline Biotech A/S, Trige, Denmark and was approved by the national authority “Danish Animal Experiments Inspectorate” (2012-15-2934-00051 C1). The animals had FELASA (Federation for Animal Science Laboratory Associations) SPF-status and the housing and changing systems were designed to assure that the SPF-status was preserved during the study. Diet (Altromin 1324) and water was administered ad libitum.

Female C.B-17 Scid female mice (nomenclature: C.B-lgh-1b Prkdcscid/lcrTac), 5–7 week of age at arrival, were supplied from Taconic-Europe, Denmark, and after 7 days of acclimatization inoculated subcutaneously with 5×10^6^ DOHH-2 cells into the right flank. Appearance of tumors was carefully monitored, and the tumors were scored (if not large enough to be measured) or measured three times a week. Tumor diameters were measured in two dimensions using a digital caliper and the volume was estimated by the following formula: L × W × ½ W (Length × Width × ½ Width). Measurements/observations began at day 0, i.e. the day of inoculation. After randomization on day 21 the animals were allocated to groups and dosed with intravenously injected vehicle (2 mL/kg, three times weekly, control group), vincristine (1 mg/kg, three times weekly, positive control), or melflufen (3 mg/kg, two times weekly, test group). Treatment continued for two weeks, and mice were terminated by cervical dislocation at the end of the experiments or at humane endpoints (maximal tumor size, weight loss of > 20 %, wounds, etc).

### Statistics

The cytotoxic IC_50_-vaules (inhibitory concentration 50 %) for the drugs were determined from log concentration-effect curves in Graph Pad Prism (GraphPad software Inc., CA, USA) using non-linear regression analysis. Statistical considerations and comparisos between melphalan and melflufen groups were based on logIC_50_-values and made with two-sided paired *t*-test, and for three groups one-way ANOVA was used. For comparison between survival curves log-rank test was used. In all tests *p* < 0.05 was considered significant.

## Results

### Cytotoxic activity in lymphoma cell lines

The cytotoxic activity of melflufen in human lymphoma cell lines is presented in Table 3. The calculated IC_50_-values varied almost 100-fold among the cell lines, identifying the DLBCL cell lines Ly-3 as the most sensitive cell line (IC_50_-values for melphalan and melflufen being 0.52 and 0.011 μM respectively) and DB as the most resistant (44 and 0.92 μM). Melflufen was consistently more potent than melphalan throughout the panel, and the average ratio of IC_50_-values was 49-fold (range 12 to 102, *p* < 0.001). The pattern of sensitivity among the cell lines was similar for all standard chemotherapeutic drugs. As expected, the correlation coefficients of log IC50-values between the alkylating agents was high (Pearson’s correlation = 0.83 for melflufen vs 4-HC, and =0.88 for melflufen vs melphalan) in this panel of lymphoma cells. Interestingly, bortezomib deviated from this pattern, and all cell lines appeared sensitive to this drug with comparably small variations in IC_50_ (range 2.0–7.1 nM).

### Cytotoxic activity in primary cultures of human lymphoma cells

**Fig. 2:**
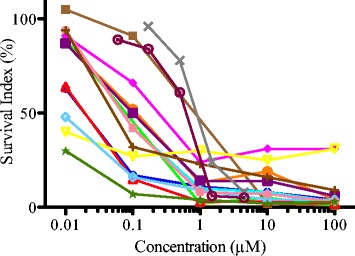
Activity of melflufen in primary lymphoma cells. The cytotoxicity of melflufen in human primary lymphoma cells, after incubation for 72 h, was tested by the Fluorometric Cytotoxicity Assay. Each dose response curve is one patient cell culture, plotted as survival index (%) as a function of concentration

Figure 2 shows the cytotoxicity of melflufen in primary human lymphoma cells, plotted as dose response curves with survival index (SI %) for each concentration tested. Sensitivity towards melflufen varied considerably (>300 fold) and the IC_50_ range from 2.7 nM to 0.55 μM. The efficacy of melflufen corresponded to a 13- to 455-fold potency improvement (*p* < 0.001) compared to melphalan (Table 4).

**Table 4:** IC_50_ (μM) for melphalan and melflufen, and melphalan/melflufen ratio in primary human lymphoma cells

Patient no	IC_50_ melphalan (μM)	IC_50_ melflufen (μM)	Ratio
1	1.7	0.018	94
2	6.7	0.51	13
3	7.0	0.059	118
4	19	0.053	363
5	89	0.55	162
6	8.5	0.12	73
7	29	0.11	271
8	6.0	0.018	337
9	2.8	<0.01	278
10	0.73	0.0080	91
11	13	0.079	160
12	1.2	0.0027	455
13	31	0.080	388
14	NA	NA	NA
15	24	0.90	27
16	16	0.66	24
MEAN	8.4	0.078	108

The potency difference between melphalan and melflufen was statistically significant (*p* < 0.001, paired 2-tailed *t*–test)

### Effect of treatment with melflufen on cell cycle phase distribution

**Fig. 3:**
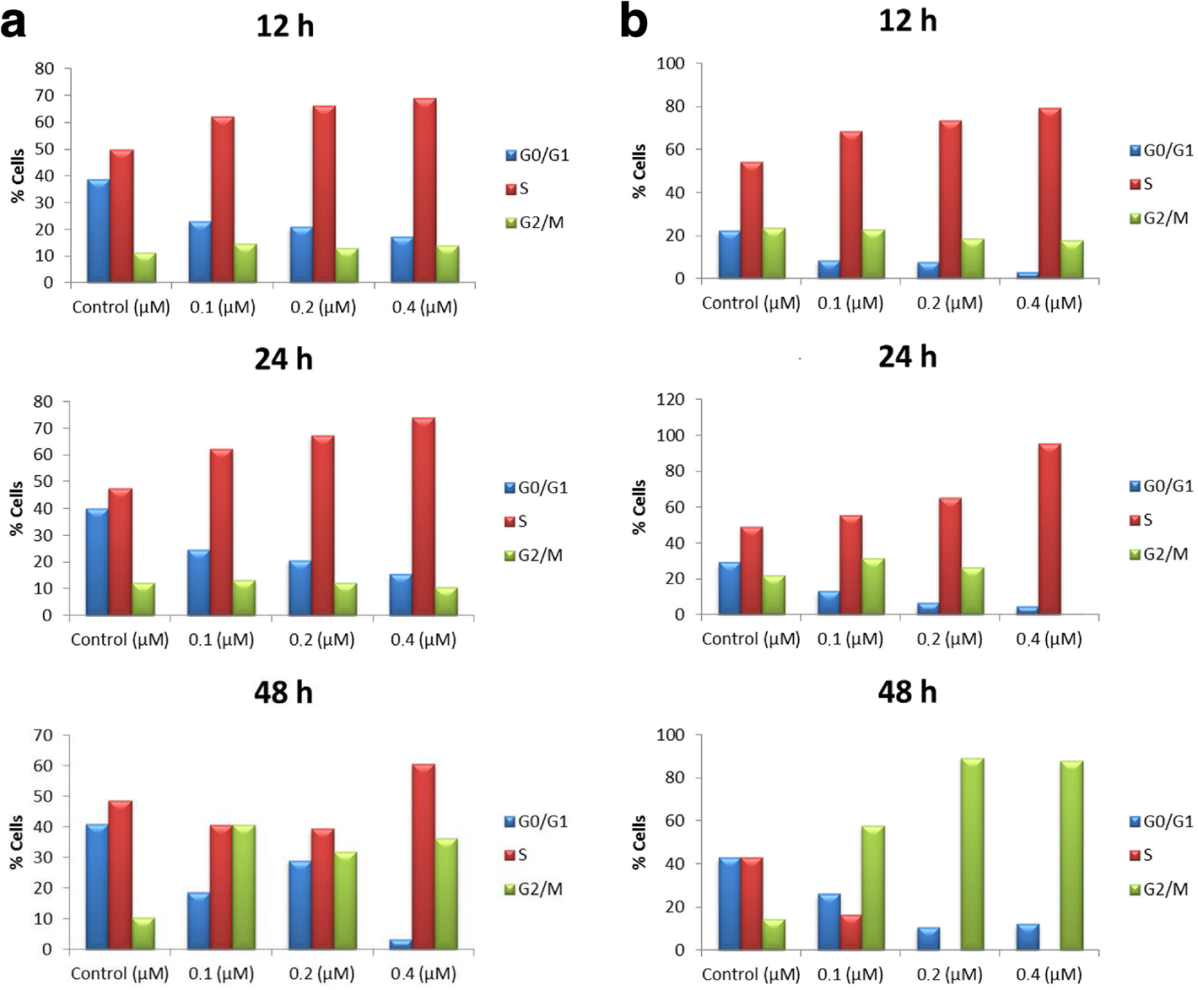
Effects of melflufen on cell cycle phase distribution. KM-H2 (**a**) and SU-DHL-10 (**b**) cell lines were incubated for 40 h during basal conditions before treatment with melflufen for 12, 24 and 48 h. The analyses were performed by flow cytometry and show distribution of the cell cycle phases G0/G1, S and G2/M resulting from treatment with melflufen 0.1-0.4 μM

The effect of treatment with melflufen on cell cycle phase distribution in KM-H2 and SU-DHL-10 cell lines was analyzed by flow cytometry and illustrates distribution of the cell cycle phases G0/G1, S and G2/M. After a 48 h treatment with melflufen, a clear accumulation of cells in the G2/M phase was seen, and this response was detected in both cell lines tested (Fig. 3). However it was much more pronounced in SU-DHL-10 cell line. In SU-DHL-10 cells, G2 arrest was already seen after 24 h of treatment with melflufen. For KM-H2 cells, 48 h were needed. In all tested concentrations a dramatic increase in G2 after 48 h of treatment was seen which is consistent with the cells trying to divide yet unable to do it due to DNA damage and thus arresting in G2.

### Activity in DOHH-2 xenografts

**Fig. 4:**
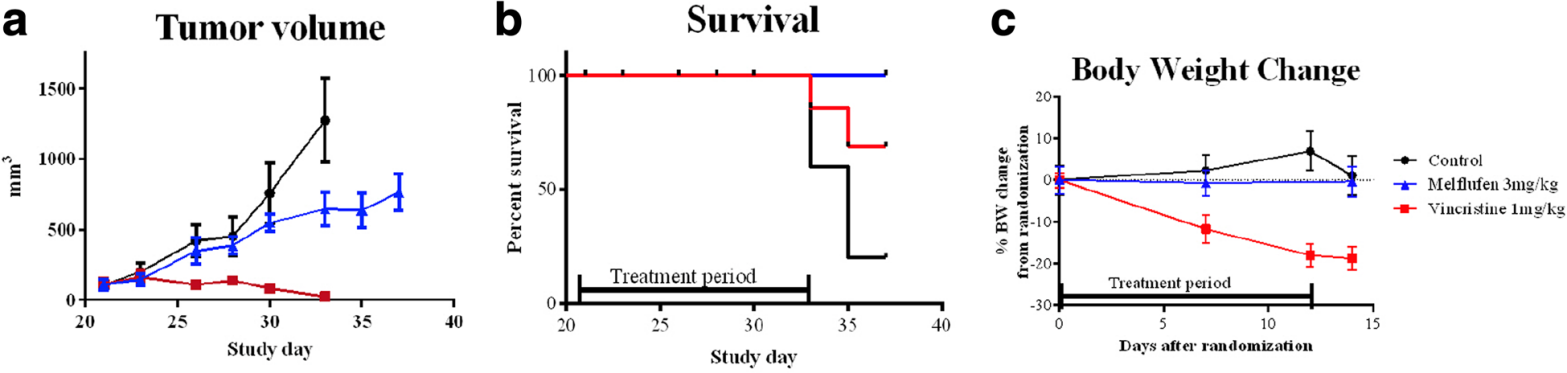
Effects of melflufen in subcutaneous DOHH-2 xenografted mice. Presented as tumor volume (**a**), survival (**b**) and body weight change (**c**). Mice in control group (*n* = 5) gained weight and showed no signs of toxicity, but individuals were prematurely sacrificed due to tumor size on day 33 (*n* = 2) and 35 (*n* = 2). Animals in vincristine control group had excellent tumor control but lost weight and one animal was sacrificed on day 25, one on day 33 due to >20 % weight reduction. Animals treated with melflufen had no weight loss but significant tumor growth reduction on day 33 (*p* < 0.05). Log-rank test showed a significant difference in survival between the control group and the melflufen treated group (*p* = 0.0144). In A and C mean values with SEM are displayed

Melflufen significantly inhibited growth of the subcutaneously xenografted DOHH-2 lymphoma tumors during the treatment period (Fig. 4a) and prolonged survival (Fig. 4b) compared to the vehicle treated control. Premature sacrifice in the vehicle treated control group but not in the melflufen group was done due to large tumors (4 of 5 animals). All remaining animals were terminated on day 37. Treatment with melflufen intravenously on a twice-weekly schedule at the dose 3 mg/kg had no detectable effect on animal health or weight development during the experiment, and all animals survived until termination. In contrast, the positive control group (treated with vincristine) presented significant body weight loss, and two animals reached the toxicity endpoint (-20 % body weight) at or before the end of the experiment (Fig. 4c).

## Discussion

Melphalan has been used for almost sixty years in the treatment of a broad spectrum of malignancies and is part of many combinations regimes. Melphalan belongs to a class of agents, i.e. alkylators, which exert cytotoxic action through covalent interaction with intracellular nucleophiles, especially DNA. Difunctional agents, able to crosslink a DNA strand within a double helix (intrastrand), between two strands (interstrand) or between DNA and proteins, are usually more active than monofunctional agents. Although covalent adduct formation is the mechanism of cytotoxicity common to all the antitumor alkylating agents, these drugs have widely different potency, toxicity and disease selectivity. Differences in the non-alkylating portions of these molecules probably lead to different biodistribution and normal tissue toxicity of these drugs [8]. New representatives of this class of drugs are continuously introduced, such as temozolomide and bendamustin.

The clinical use of most chemotherapeutic agents is limited by the associated toxicity, often originating from organs with high proliferation (e.g. bone marrow). Many attempts have been made to, more or less, specifically target the tumor cells or the tumor microenvironment, for example, the use of a prodrug approach where the prodrug is selectively activated in tumors overexpressing the prodrug-activating enzyme(s) [32]. Many such targets have been suggested in the literature, among them different aminopeptidases like APN, which is described as overexpressed in many malignant tissues making it a possible and attractive target for cancer chemotherapy [14]. Melflufen is a dipeptide derivative carrying melphalan as one of its amino acid moieties. Compelling evidence shows that the activity benefit of melflufen compared to melphalan originates from APN dependent intracellular cleavage of melflufen yielding high concentrations of melphalan in the cytoplasm, i.e. a targeted delivery [14, 16, 22].

APN/CD13 is commonly expressed in hematopoietic malignancies of myelomonocytic origin and has less commonly been described in lymphoid neoplasms. However, effects of the aminopeptidase inhibitor bestatin on lymphoma cells have been shown in vitro [26], and the effects of this drug in lymphoma patients have also been evaluated in clinical trials [26, 33]. Wickstrom et al. [18] investigated the effects of melflufen in primary cultures of tumor cells from 176 patients with various diagnoses. Among these were fourteen patients with lymphoma, and the in vitro activity difference between melphalan and melflufen was in this diagnosis exceptionally high (160×) prompting further investigation of the activity of melflufen in lymphoma.

The results presented here show that melflufen is indeed an active drug both in Hodgkin and non-Hodgkin lymphoma cell lines in vitro, yielding IC_50_-values in the range of 11 nM to 0.92 μM. A similar pattern was found in patient cells (IC_50_ of 2.7 nM to 0.55 μM) clearly suggesting that lymphoma is a possible clinical diagnosis for future use of melflufen. The in vivo xenograft model study indicated modest activity of melflufen in the dose and regimen used (3 mg/kg on a twice weekly schedule), but on the other hand, without any signs of toxicity the dose and/or intensity of the treatment schedule could most probably be increased. Animals in the positive control group suffered severely from toxicity, and albeit excellent tumor control, some of them had to be prematurely sacrificed due to treatment associated weight loss.

## Conclusion

The conclusion of this study demonstrates that melflufen is an active drug against lymphoma in vitro and in vivo and further evaluation in this diagnosis seems warranted.

### Ethics approval and consent to participate

The xenograft study was performed at Pipeline Biotech A/S, Trige, Denmark and was approved by the national authority “Danish Animal Experiments Inspectorate” (2012-15-2934-00051 C1).

The use of patient samples was approved by the regional Ethics Committee of Uppsala University (Ns 2008/246 and 2014/233).

The use of the patients’ samples in research was approved without written informed consent of the patients. The reasons why the ethical committee waived informed consent were that the patients had approved storing of samples in the Uppsala biobank and that these samples could be used for research after ethical approval without contacting the patient if the committee found the research of good quality and not compromising integrity of the patient. Furthermore, all patients included in this study have given an oral informed consent but that was not a requirement of the ethical committee.

### Availability of data and materials

The datasets supporting the conclusions of this article are included within the article.

## Abbreviations

HL: Hodgkin lymphoma
DLBCL: Diffuse large B-cell lymphoma
AML: Acute myeloid leukemia
DMSO: Dimethylsulfoxide
PBS: Phosphate buffered saline
4-HC: 4-hydroxy-cyclophosphamide
FMCA: Fluorometric microculture cytotoxicity assay
FDA: Fluorescein diacetate
SI: Survival index
PI: Propidium iodide
MCL: Mantle cell lymphoma
NHL: Non-Hodgkin lymphoma
T-LB: Lymphoblastlymphoma of T-cell type
FL: Follicular lymphoma
T-PLL: Prolymphocytic leukemia of T-cell type
DLBCLtr GC: Diffuse large B-cell lymphoma transformed from follicular lymphoma of germinal centre subtype
Nos: Not otherwise specified
